# Functional polymorphisms in the promoter region of miR-17-92 cluster are associated with a decreased risk of colorectal cancer

**DOI:** 10.18632/oncotarget.19753

**Published:** 2017-07-31

**Authors:** Ruifen Sun, Yundan Liang, Fang Yuan, Xinwen Nie, Hong Sun, Yanyun Wang, Tao Yu, Linbo Gao, Lin Zhang

**Affiliations:** ^1^ Laboratory of Molecular and Translational Medicine, West China Institute of Women and Children’s Health, Key Laboratory of Birth Defects and Related Diseases of Women and Children (Sichuan University), Ministry of Education, West China Second University Hospital, Sichuan University, Chengdu, Sichuan 610041, P.R. China; ^2^ Central Laboratory, Yunnan University of Chinese Traditional Medicine, Kunming 650500, Yunnan, P.R. China; ^3^ Department of Pathology and Pathophysiology, Chengdu Medical College, Chengdu, Sichuan 610083, P.R. China; ^4^ Division of Reproductive Medical Center, West China Second University Hospital, Sichuan University, Chengdu, Sichuan 610041, P.R. China; ^5^ Department of Child Health, West China Second University Hospital, Sichuan University, Chengdu, Sichuan 610041, P.R. China

**Keywords:** miR-17-92, promoter, polymorphism, luciferase activity, colorectal cancer

## Abstract

miR-17-92 cluster is identified as a potential oncogenic miRNA. The aim of this study was to investigate the association of polymorphisms in the promoter region of miR-17-92 cluster with the risk of colorectal cancer (CRC). Three polymorphisms (i.e., rs9588884, rs982873 and rs1813389) in the promoter of miR-17-92 were analyzed among 874 cases and 1132 controls using a TaqMan allelic discrimination assay or a polymerase chain reaction-restriction fragment length polymorphism method. Relative expression of miR-17-92 was examined among CRC tumors and noncancerous tissues using quantitative reverse transcription-PCR. Transcriptional activities were measured using dual-luciferase reporter assay. We found a significantly reduced CRC risk with the rs9588884 (GG vs. CC: adjusted OR = 0.46, 95% CI, 0.35-0.62; dominant model: adjusted OR = 0.72, 95% CI, 0.59-0.86; recessive model: adjusted OR = 0.53, 95% CI, 0.40-0.69) and the rs982873 (CC vs. TT: adjusted OR = 0.60, 95%CI, 0.46-0.80; recessive model: adjusted OR = 0.62, 95% CI, 0.49-0.80). Haplotype analysis showed that the GCG haplotype had a decreased risk for CRC compared to the CTA haplotype (adjusted OR = 0.67, 95% CI, 0.57-0.79). The rs9588884 GG displayed a lower level of miR-20a and the rs982873 CC displayed a lower level of miR-17. Additionally, the rare allele of rs9588884 G and the rs982873 C revealed a reduced luciferase activity. These findings indicate that the rs9588884 GG and the rs982873 CC in the promoter of miR-17-92 may protect against CRC, possibly by decreasing transcriptional activity and eventually resulting in lower levels of miR-20a and miR-17.

## INTRODUCTION

microRNAs (miRNAs) are a class of non-coding RNAs, ∼23 nucleotides in length, which function as post-transcriptional gene regulators by repressing protein translation or accelerating degradation of target messenger RNAs (mRNAs) [1, 2]. It has been noticed that some miRNAs show high similarity in sequence and are clustered [1]. A cluster of miRNAs is defined as miRNAs closer to each other in the genome that are initially transcribed into one long primary transcripts and subsequently cleaved into shorter individual precursor miRNAs [3]. Approximately 30% of clustered miRNAs are transcribed as polycistrons and display analogous expression patterns [1, 3–6]. Transcription of clustered miRNAs may be regulated either by their common promoter or by a host gene promoter [7, 8].

miR-17-92 cluster, one of the best characterized polycistronic miRNAs, located in an intron of non-protein coding gene *MIR17HG* (miR-17-92 cluster host gene) on chromosome 13 in the human genome. The primary transcript of miR-17-92 spans 800 nucleotides within its host gene, yielding six mature miRNAs: miR-17, miR-18a, miR-19a, miR-20a, miR-19b-1 and miR-92a-1 [9, 10]. This genomic locus, previously known as chromosome 13 open reading frame 25, is a target for 13q31-q32 amplification in a large spectrum of human cancers including colorectal cancers (CRC) [11, 12]. All members of miR-17-92 cluster except for miR-18a were overexpressed in CRC, pointing to a key role of miR-17-92 cluster in CRC carcinogenesis [13–20].

It is widely recognized that some single nucleotide polymorphisms (SNPs) in the promoter region of coding genes are functional by influencing transcriptional activity. Recent evidence has showed that SNPs in the promoter of miRNAs may alter miRNA function and/or expression, and eventually affect individuals’ susceptibility to cancer, including CRC [21–26]. One example is rs4705342 T>C in the promoter region of miR-143. The rs4705342 TC/CC genotypes had a 0.68-fold decreased risk of prostate cancer and the risk-associated T allele increased the protein-binding affinity and reduced the promoter activity [21]. Another example is rs767649 in the promoter of miR-155. The rs767649 TT genotype was associated with a significantly reduced risk of cervical cancer, possibly by downregulating the expression of miR-155 at the transcriptional level [22]. Previously, the core-promoter of miR-17-92 cluster has been experimentally identified [27, 28]. In this study, we aimed to investigate the association between SNPs in the promoter of miR-17-92 cluster and risk of CRC in a Chinese population.

## RESULTS

### Characteristics of the study population

Of all the samples analyzed, 1132 controls and 874 cases were successfully genotyped. Age and gender distributions were similar between cases and controls. Well-moderately differentiated tumor was present in 531 of the CRC patients, while poorly-undifferentiated tumor in 343 patients. There were 58.6% clinical stage I-II patients and 41.4% clinical stage III-IV patients. There were 33.1% patients with lymph node metastasis and 66.9% patients without lymph node metastasis (Table 1).

**Table 1:** Characteristics of the study population

Variables	Controls (n = 1132)	Patients with CRC (n = 874)
Age (years, mean ± SD)	59.1 (± 12.1)	60.7 (± 13.4)
Gender (%)		
Male	641 (56.6)	526 (60.2)
Female	491 (43.4)	348 (39.8)
Differentiated status (%)		
Well-Moderately		531 (60.8)
Poorly-Undifferentiated		343 (39.2)
Clinical stage (%)		
I- II		512 (58.6)
III- IV		362 (41.4)
Lymph node metastasis (%)		
Yes		289 (33.1)
No		585 (66.9)

CRC, colorectal cancer; SD, standard deviation.

### Rs9588884 and rs982873 in the promoter of miR-17-92 cluster protect against CRC risk

The association between the 3 SNPs (i.e., rs9588884, rs982873 and rs1813389) in the promoter of miR-17-92 cluster and CRC risk is shown in Table 2. The genotype distributions in controls were concordant with the HWE (*P* > 0.05). The rs9588884 GG and CG/GG genotypes were associated with a decreased risk of CRC (GG vs. CC: adjusted OR = 0.46, 95% CI, 0.35-0.62, *P* < 0.001; dominant model: adjusted OR = 0.72, 95% CI, 0.59-0.86, *P* < 0.001; recessive model: adjusted OR = 0.53, 95% CI, 0.40-0.69, *P* < 0.001). The rare homozygous genotype of rs982873 was also found to be associated with a reduced risk of CRC (CC vs. TT: adjusted OR = 0.60, 95%CI, 0.46-0.80, *P* < 0.001; recessive model: adjusted OR = 0.62, 95% CI, 0.49-0.80, *P* < 0.001). The association was not found between the rs1813389 and CRC risk.

**Table 2:** Association between the polymorphisms in the promoter of miR-17-92 and risk of CRC

SNPs	Controls (n = 1132) (%)	CRC (n = 874) (%)	Adjusted OR (95% CI) †	*P* value
rs9588884				
CC	350 (30.9)	334 (38.2)	1.00 (Ref)	
CG	580 (51.2)	450 (51.5)	0.80 (0.66-0.98)	0.03
GG	202 (17.8)	90 (10.3)	0.46 (0.35-0.62)	< 0.001
Dominant model			0.72 (0.59-0.86)	< 0.001
Recessive model			0.53 (0.40-0.69)	< 0.001
rs982873				
TT	330 (29.2)	280 (32.0)	1.00 (Ref)	
TC	582 (51.4)	481 (55.0)	0.98 (0.80-1.20)	0.83
CC	220 (19.4)	113 (12.9)	0.60 (0.46-0.80)	< 0.001
Dominant model			0.88 (0.73-1.06)	0.15
Recessive model			0.62 (0.49-0.80)	< 0.001
rs1813389				
AA	371 (32.8)	295 (33.8)	1.00 (Ref)	
AG	577 (51.0)	462 (52.9)	1.02 (0.83-1.24)	0.88
GG	184 (16.3)	117 (13.4)	0.81 (0.61-1.06)	0.13
Dominant model			0.96 (0.80-1.16)	0.70
Recessive model			0.80 (0.62-1.03)	0.08

CRC, colorectal cancer; SNP, single nucleotide polymorphism; OR, odd ratio; CI, confidence interval.

† adjusted by age and gender.

After stratification analyses according to clinical parameters, such as differentiated status, clinical stage and lymph node metastasis, we found that patients carrying the rs982873 TC genotype were less likely to develop clinical stage III-IV CRC (TC vs. TT: adjusted OR = 0.69, 95% CI, 0.51-0.93, *P* = 0.02). Additionally, patients with rs1813389 GG genotype had a reduced risk of developing clinical stage III-IV (GG vs. AA: adjusted OR = 0.58, 95% CI, 0.37-0.92, *P* = 0.02) and lymph node metastasis (GG vs. AA: adjusted OR = 0.57, 95% CI, 0.35-0.95, *P* = 0.02). No significant association, however, was observed between the rs9588884 and clinical features of CRC (Supplementary Tables 1-3).

We then did haplotype analysis to test whether any potential haplotype was associated with the risk of CRC. Analysis by the SHEsis program showed that the 3 SNPs were in moderate LD (rs9588884-rs982873: D’ = 0.579, r^2^ = 0.298; rs982873-rs1813389: D’ = 0.496, r^2^ = 0.225; rs9588884-rs1813389: D’=0.709, r^2^ = 0.488) and more than 6 haplotypes were identified. As shown in Table 3, compared to the most common CTA haplotype, the GCG haplotype had a decreased risk for CRC (adjusted OR = 0.67, 95% CI, 0.57-0.79, *P* < 0.001).

**Table 3:** Haplotype analysis of rs9588884-rs982873-rs1813389 between cases and controls

Haplotype †	Controls (%)	CRC (%)	OR (95% CI)	*P* value
CTA	1017 (44.9)	697 (39.9)	1.00 (Ref)	
GCG	711 (31.4)	327 (18.6)	0.67 (0.57-0.79)	< 0.001
CCA	220 (9.7)	140 (8.0)	0.93 (0.74-1.17)	0.53
GCG	182 (8.0)	131 (7.5)	1.05 (0.82-1.34)	0.69
GCA	82 (3.7)	78 (4.5)	1.39 (1.00-1.92)	0.05

† indicates haplotype frequencies greater than 3%.

CRC, colorectal cancer; OR, odd ratio; CI, confidence interval.

### Rs9588884 and rs982873 rare genotype associates the expression of miR-17-92

**Figure 1:**
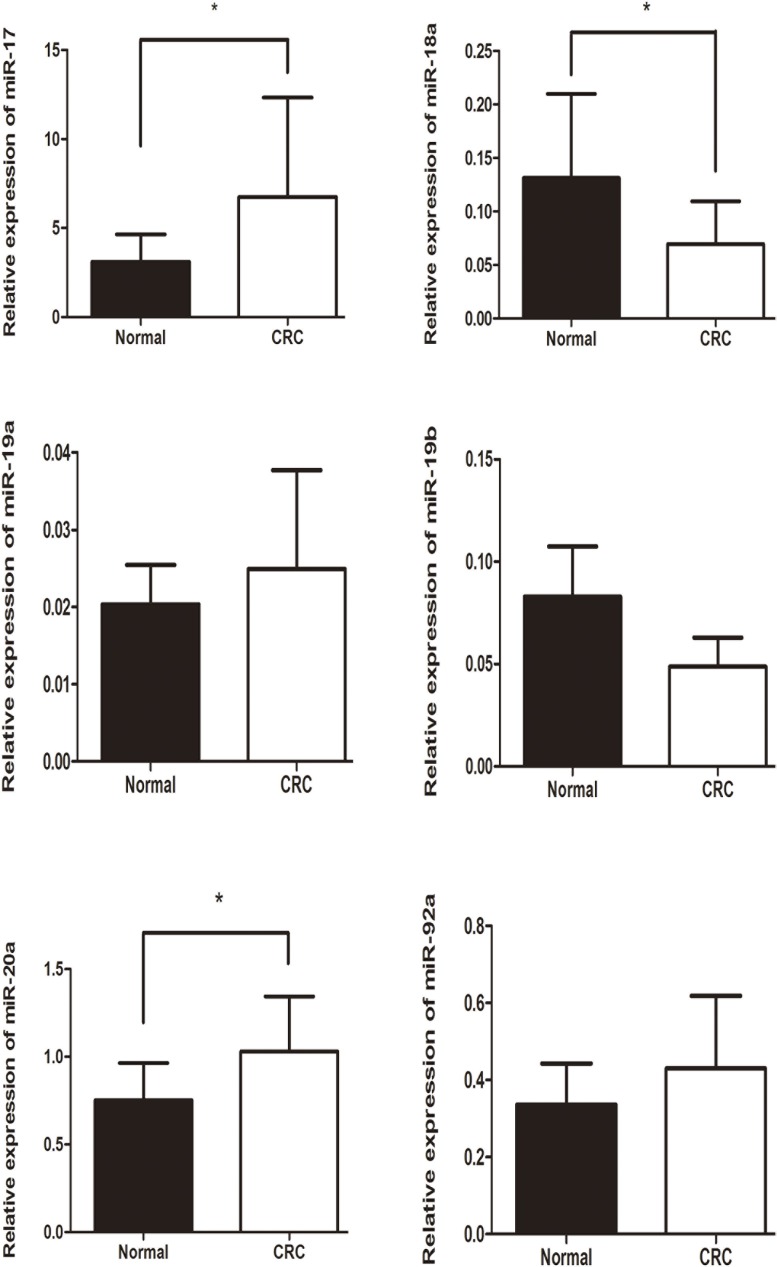
Relative expression of miR-17-92 cluster among CRC tissues and paracancerous normal tissues Data was presented as mean ± standard error (* *P* < 0.05).

**Figure 2:**
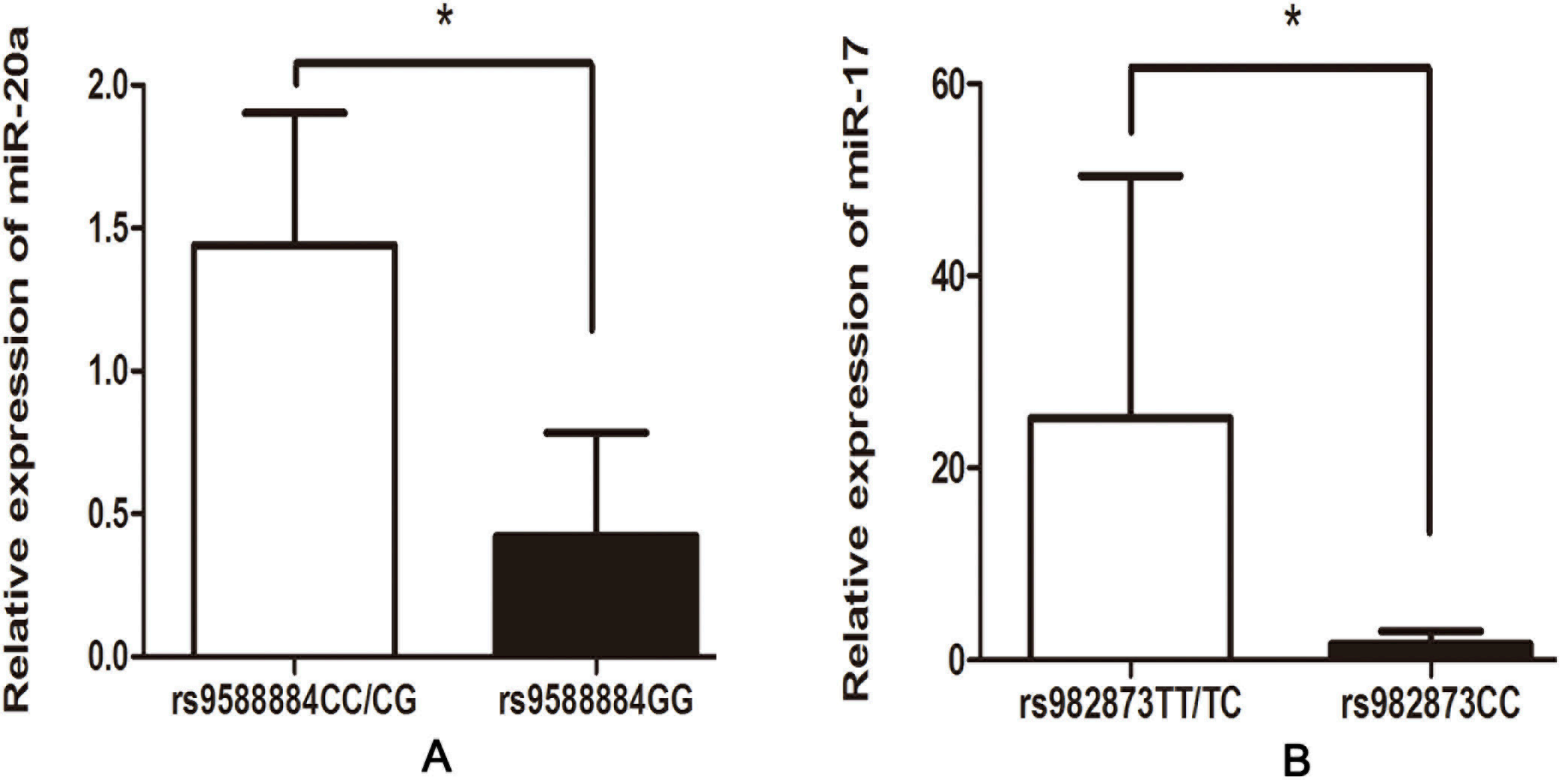
Correlation between the SNPs in the promoter of miR-17-92 cluster and their expression in CRC tissues **(A)** rs9588884 and miR-20a; **(B)** rs982873 and miR-17 (* *P* < 0.05).

Based on the positive data above, we next assessed whether the rs9588884 and rs982873 influence the expression of miR-17-92. Relative expression of miR-17-92 cluster was examined among CRC tumors and noncancerous tissues. As shown in Figure 1, levels of miR-17 and miR-20a were upregulated in CRC tissues (*P* = 0.03 and 0.016, respectively), whereas miR-18a level was downregulated in CRC tissues (*P* = 0.04). Notably, after analyzing relevancy of the rs9588884 and rs982873 and expression of miR-17-92 cluster, we found that the rare genotype of rs9588884 GG displayed a lower level of miR-20a (*P*=0.016) and rs982873 CC displayed a lower level of miR-17 (*P*=0.006) (Figure 2).

### Effects of SNPs in the promoter of miR-17-92 cluster on the transcriptional activity

**Figure 3:**
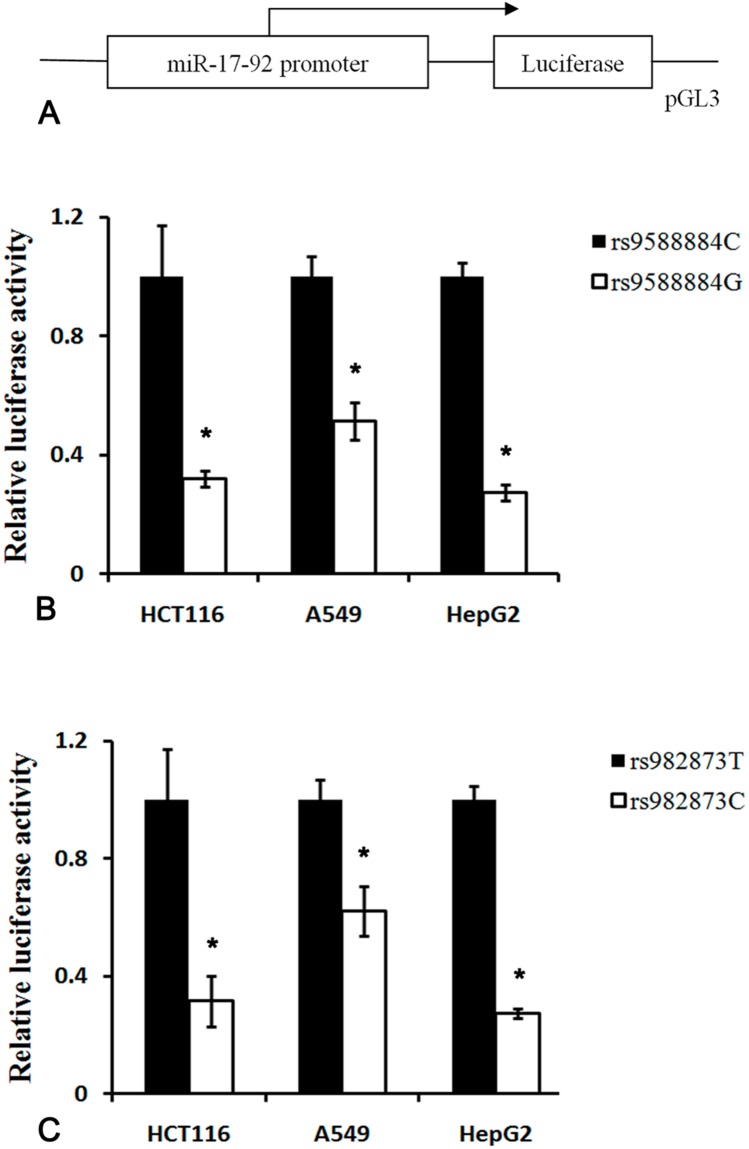
Luciferase reporter assay of the SNPs in the promoter of miR-17-92 cluster **(A)** Schematic representation of the promoter of miR-17-92 into pGL3 vector; **(B)** the rs9588884 G allele displayed a lower transcriptional activity compared to the rs9588884 C allele; **(C)** the rs982873 C allele exhibited a lower transcriptional activity compared to the rs982873 T allele (* *P* < 0.05).

Given the rare genotype of the rs9588884 and rs982873 protecting against CRC risk, we did further analysis evaluating the effect of the rs9588884 and rs982873 on the transcriptional activity. As shown in Figure 3, the rs9588884 G allele exhibited a lower transcriptional activity than the rs9588884 C allele (*P* < 0.01). The decreased luciferase activity was also observed in the rs982873 C allele when compared to its counterpart (rs982873 T allele) (*P* < 0.01).

## DISCUSSION

To the best of our knowledge, this is the first study to investigate the association between SNPs in the promoter of miR-17-92 and CRC risk. A reduced CRC risk was observed in individuals with either the rs9588884 GG or the rs982873 CC genotype. The decreased CRC risk was also observed in individuals with GCG haplotype. Additionally, the rare allele of rs9588884 G and the rs982873 C revealed a reduced luciferase activity and a lower level of miR-20a and miR-17, respectively. These findings indicate that the rs9588884 GG and the rs982873 CC in the promoter of miR-17-92 may protect against CRC in a Chinese population.

Regarding genetic contributions to CRC susceptibility, previous association studies focused on coding genes [29]. Recently, accumulating epidemiological studies evaluated the association between miRNA related polymorphisms and CRC risk [24-26, 30, 31]. Our previous work showed an rs4938723 in the promoter of miR-34b/c having a 0.56-fold decreased risk of CRC [24]. Our finding was confirmed by subsequent case-control study and meta-analysis [26, 32, 33]. In this study, we investigated the distributions of three SNPs in the promoter of miR-17-92 (i.e., rs9588884, rs982873 and rs1813389) among 874 CRC patients and 1132 age- and gender-matched controls. We found that the rs9588884 GG, rs982873 CC and GCG haplotype were associated with a reduced risk of CRC. As all the samples are Han Chinese, validation studies in different ethnicities are required.

miR-17-92 cluster, firstly described by He and colleagues, has been identified as a potential oncogenic miRNA [9]. Overexpression of miR-17-92 cluster is not only involved in the progression of colorectal adenoma to carcinoma but also related to poor survival of CRC [34, 35]. Upregulation of miR-92 in plasma may be used as a non-invasive molecular marker for CRC screening, with a sensitivity of 89% and a specificity of 70% [36]. Repression of miR-17 and miR-92a induces apoptosis after DNA damage and re-introduction of miR-17 and miR-20a promotes proliferation and invasion of CRC cells by targeting gamma-amino-butyric acid type B receptor 1 [13, 37, 38]. Knockdown of miR-19a in CRC cells with compromised APC function reduces cell growth, migration and invasion and enforced expression of miR-19a overrides APC tumor suppressor activity [39]. Exogenous miR-20a reduces the expression of MYC-regulating genes and abrogates the TGF-β-induced repression of MYC promoter activity [40]. MYC is a crucial mediator of miR-17-92 promoter activity and expression [35, 41, 42]. In mouse tumor model, high levels of miR-17-92 inhibit tumor growth and metastasis [43]. However, in transgenic mice, overexpression of miR-17-92 inhibits tumor growth and tumor angiogenesis by targeting multiple genes, such as transforming growth factor β type II receptor, hypoxia induced factor-1α and vascular endothelial growth factor A [44]. Additionally, the 13q31 amplicon, containing miR-17-92 precursor, is related to status of lymph node metastases in CRC [45]. These findings point out a pivotal role of miR-17-92 in CRC development.

In this study, we examined the expression of miR-17-92 cluster among CRC tumors and noncancerous tissues. We found that levels of miR-17 and miR-20a were upregulated and miR-18a level was downregulated in CRC tissues, which were consistent with previous reports [13-19, 46]. These findings indicate that miR-17 and miR-20a may function as oncogenes. Notably, we found that the rare genotype of rs9588884 GG and rs982873 CC exhibited a lower level of miR-20a and miR-17, respectively. Moreover, the rare allele of rs9588884 G and the rs982873 C revealed a lower luciferase activity compared to their counterparts. Taken together, the rs9588884 G and the rs982873 C may reduce transcriptional activity, decrease levels of miR-20a and miR-17, and finally lead to a lower risk for developing CRC. This may be a plausible mechanism for explaining the protective effect of SNPs in the promoter of miR-17-92 cluster on CRC risk.

This study has large sample size and the genotype frequencies were in HWE. These benefits, however, cannot override the limitation of this study. Data were not available for some risk factors such as diet, alcohol intake, and cigarette consumption, which prevented our further gene-environment interaction analysis. Due to lack of sufficient DNA quantity, *KRAS* and *APC* mutation cannot be examined. Without detailed survival information of CRC patients, the effect of the SNPs in the promoter of miR-17-92 on the outcome of CRC cannot be assessed. More detailed data will help to create a comprehensive understanding of the 3 SNPs in CRC tumorigenesis.

In conclusion, this study provides the first evidence that the rs9588884 GG and the rs982873 CC in the promoter of miR-17-92 cluster protect against CRC development, possibly by decreasing transcriptional activity and eventually resulting in lower levels of miR-20a and miR-17. Further studies are of great importance to confirm these results, especially in diverse ethnic groups.

## MATERIALS AND METHODS

### Study subjects

A hospital-based case-control study was conducted to evaluate SNPs in the promoter of miR-17-92 cluster in relation to CRC risk. The analysis was performed on genomic DNA from 966 CRC cases together with 1154 controls. Detailed information of study population is described in our previous studies [30, 31]. In brief, CRC samples were enrolled from the West China Hospital of Sichuan University, the Luoyang Central Hospital Affiliated to Zhengzhou University and the Affiliated Hospital of North Sichuan Medical College between January 2010 and February 2015. The diagnosis of CRC was confirmed by histopathological analysis. Clinical data was retrieved by retrospectively reviewing surgical and pathological records, including age, gender, ethnicity, differentiated status, tumor size, lymph node metastasis and distant metastasis. Clinical stage was determined according to TNM classification system of the Union for International Cancer Control. We excluded those patients with an evidence of personal or family history of inflammatory diseases or cancer in the intestine.

The controls were healthy volunteers and selected from the same hospital during the same period. They were frequency-matched to the cases based on age, gender, living area and ethnicity. Individuals with previous diagnosis of inflammatory diseases or cancer in the intestine were excluded. All participants were unrelated Han population living in the central or western area of China. The study was approved by the Ethics Committee of the West China Second University Hospital and all subjects agreed to participate in the study.

### SNPs selection and genotyping

According to location of transcription start site (TSS) of miR-17-92 [27, 28], we analyzed the region -2.0kb upstream from the TSS using UCSC genome browser (http://genome.ucsc.edu/). SNPs with minor allele frequency greater than 10% in Chinese Han population were selected. Finally, three SNPs were identified, i.e., rs9588884, rs982873 and rs1813389, which located in the -1202, -591 and -252bp upstream from the TSS, respectively.

Genomic DNA was isolated by using commercial extraction kits (Bioteke, Beijing, China and Qiagen, Hilden, Germany) according to the instruction manual. The rs9588884 was genotyped by using a TaqMan allelic discrimination method. The rs982873 and rs1813389 were genotyped by using a polymerase chain reaction-restriction fragment length polymorphism assay. Common Taq was used for DNA amplification. The primer sequences were as follows: for rs982873, 5’-TGTCCGGCAATCATGAAGTA-3’ (forward) and 5’-GCTGTATTACGTCTGGAAAGTGCC-3’ (reverse); for rs1813389, 5’-CCAGGGTAAGG CTCCATACAT-3’ (forward) 5’-AAAGCTTTTCTGACACTTTGAGTAGT-3’ (reverse). PCR products were digested with *Bsl* I and *Hinc* II, respectively (New England BioLabs, Beverly, MA). The rs982873 T allele remained intact of 216 bp, whereas the rs982873 C allele was cut into two smaller fragments of 190 and 26 bp. The rs1813389 A allele remained intact of 172 bp, whereas the rs1813389 G allele was cut into two smaller fragments of 145 and 27 bp. The genotyping analysis was performed by two laboratory staff independently, both of whom were blinded to the case–control status of each sample. About 5% samples were regenotyped by DNA Sanger sequencing (TsingKe, Chengdu, China) and the results of the both methods agreed with each other.

### Quantitative reverse transcription-PCR assay

RNA was extracted from CRC tumors and noncancerous tissues using TriPure Isolation Reagent according to the manufacturer’s instructions (Roche, Indianapolis, IN). For examination of miR-17-92, 1 μg of RNA was converted to cDNA using a commercial kit according to the manufacturer’s manual (Ribobio, Guangzhou, China). Commercial primer sets of miR-17-92 were obtained from Ribobio company, Guangzhou, China. Quantitative real-time PCR was performed using Eppendorf MasterCycler RealPlex with SYBR green Master Mix (Qiagen, Hilden, Germany). Data were normalized using U6 snRNA as an internal control and relative expression of miR-17-92 was quantified using 2^-ΔCt^ method [47].

### Plasmid construction and dual-luciferase reporter assay

The promoter region of miR-17-92 cluster containing rs9588884 C and rs982873 T was synthesized by PCR (from -1245 to +77 bp) using high fidelity Taq and the following primers: 5’-TCTGGTACCTTGCACTCCTGGGATGAAAT-3’ (forward) and 5’- CTAGAGCTCCTTTGTCACTATGACAGGAGCA-3’ (reverse). After digestion with *Kpn* I and *Sac* I, the DNA fragment was inserted into pGL3 vector (Promega, Madison, WI). Mutation constructs of rs9588884 G and rs982873 C were created by using a QuickChange Site-Directed Mutagenesis kit (Stratagene, La Jolla, CA). The insert sequences were verified by DNA sequencing.

HCT116, A549 and HepG2 cells were derived from a stock maintained in our laboratory. The cells were routinely cultured in Dulbecco’s modified Eagle’s medium supplemented with 10% fetal bovine serum and 1% penicillin/streptomycin. The cells were seeded in 24-well plates (1 × 10^5^/well) for 24h, then transfected with 2 μg wild type and mutant type plasmids using X-treme GENE HP reagent (Roche, Indianapolis, IN). Renilla luciferase reporter vector was used as an internal control to normalize the promoter activity. Firefly and renilla luciferase activities were measured consecutively by using the dual-luciferase assay system (Promega) 48h after transfection. Relative luciferase activity was calculated as the ratio of firefly to renilla activity.

### Statistical analysis

Statistical analysis was performed using SPSS statistics program (v.19.0; SPSS Inc., Chicago, IL). Mean (± standard deviation) of continuous data and frequencies of categorical data were calculated. A free software based on chi-square test was used to measure Hardy-Weinberg equilibrium (HWE). The associations between the 3 SNPs (i.e., rs9588884, rs982873 and rs1813389) in the promoter of miR-17-92 cluster and CRC risk were assessed by computing odds ratio (OR) and respective 95% confidence interval (CI). The heterozygous comparison, homozygote comparison, dominant model and recessive model were analyzed by unconditional logistic regression. OR was adjusted by age and gender. Linkage disequilibrium (LD), coefficient (D’ and r^2^) for haplotypes and the distributions were performed using SHEsis software [48]. According to Bonferroni correction, statistical significance for each genotype comparison was set at *P* < 0.025. In haplotype analysis, Bonferroni-corrected α-level of 0.005 (0.05/15) was considered statistically significant. Relative ludiferase activity between two constructs was examined using Student’s *t* test. Relative expression of miR-17-92 and the relationship with SNPs were evaluated using Mann–Whitney U test.

## SUPPLEMENTARY MATERIALS TABLES


