# Supplementary material for: Functional polymorphisms in the promoter region of miR-17-92 cluster are associated with a decreased risk of colorectal cancer
Source: Oncotarget. 2017 Jul 31;8(47):82531–40. doi: 10.18632/oncotarget.19753 (PMC5669908; doi:10.18632/oncotarget.19753)
Supplement: Supplementary file 1 [file oncotarget-08-82531-s001.pdf]

Functional polymorphisms in the promoter region of miR-17-92 cluster are associated with a decreased risk of colorectal cancer

SUPPLEMENTARY MATERIALS

Supplementary Table 1: Stratified analyses of the rs9588884 with clinical features of CRC

Clinical features			Adjusted OR (95% CI) †	*P* value
Differentiated status	Well-Moderately	Poorly-Undifferentiated		
CC	191 (36.0)	143 (41.7)	1.00 (Ref)	
CG	286 (53.9)	164 (47.8)	0.77 (0.58-1.03)	0.08
GG	54 (10.2)	36 (10.5)	0.88 (0.55-1.42)	0.61
Dominant model			0.79 (0.60-1.04)	0.10
Recessive model			1.03 (0.66-1.62)	0.88
Clinical stage	I-II	III-IV		
CC	186 (36.3)	148 (40.9)	1.00 (Ref)	
CG	270 (52.7)	180 (49.7)	0.85 (0.64-1.13)	0.27
GG	56 (10.9)	34 (9.4)	0.77 (0.47-1.25)	0.29
Dominant model			0.83 (0.63-1.11)	0.21
Recessive model			0.84 (0.53-1.32)	0.44
Lymph node metastasis	Yes	No		
CC	116 (40.1)	218 (37.3)	1.00 (Ref)	
CG	144 (49.8)	306 (52.3)	1.11 (0.82-1.51)	0.49
GG	29 (10.0)	61 (10.4)	1.13 (0.68-1.88)	0.65
Dominant model			1.12 (0.83-1.50)	0.47
Recessive model			1.06 (0.66-1.71)	0.81

CRC, colorectal cancer; OR, odds ratio; CI, confidence interval.
† adjusted by age and gender.

Supplementary Table 2: Stratified analyses of the rs982873 with clinical features of CRC

**Clinical features**			**Adjusted OR (95% CI) †**	***P* value**
Differentiated status	Well-Moderately	Poorly-Undifferentiated		
TT	177 (33.3)	103 (30.0)	1.00 (Ref)	
TC	292 (55.0)	189 (55.1)	1.11 (0.82-1.50)	0.50
CC	62 (11.7)	51 (14.9)	1.41 (0.90-2.20)	0.13
Dominant model			1.16 (0.86-1.55)	0.33
Recessive model			1.31 (0.88-1.95)	0.19
Clinical stage	I-II	III-IV		
TT	150 (29.3)	130 (35.9)	1.00 (Ref)	
TC	298 (58.2)	183 (50.6)	0.69 (0.51-0.93)	0.02
CC	64 (12.5)	49 (13.5)	0.85 (0.54-1.33)	0.46
Dominant model			0.72 (0.54-0.96)	0.03
Recessive model			1.07 (0.71-1.60)	0.75
Lymph node metastasis	Yes	No		
TT	98 (33.9)	182 (31.1)	1.00 (Ref)	
TC	149 (51.6)	332 (56.8)	1.23 (0.90-1.69)	0.20
CC	42 (14.5)	71 (12.1)	0.97 (0.61-1.54)	0.90
Dominant model			1.08 (0.87-1.60)	0.30
Recessive model			0.84 (0.55-1.28)	0.42

CRC, colorectal cancer; OR, odds ratio; CI, confidence interval.
† adjusted by age and gender.

Supplementary Table 3: Stratified analyses of the rs1813389 with clinical features of CRC

**Clinical features**			**Adjusted OR (95% CI) †**	***P* value**
Differentiated status	Well-Moderately	Poorly-Undifferentiated		
AA	179 (33.7)	116 (33.8)	1.00 (Ref)	
AG	272 (51.2)	190 (55.4)	1.08 (0.80-1.46)	0.61
GG	80 (15.1)	37 (10.8)	0.71 (0.45-1.12)	0.14
Dominant model			1.00 (0.75-1.33)	0.99
Recessive model			0.68 (0.45-1.03)	0.06
Clinical stage	I-II	III-IV		
AA	159 (31.1)	136 (37.6)	1.00 (Ref)	
AG	275 (53.7)	187 (51.7)	0.79 (0.59-1.07)	0.13
GG	78 (15.2)	39 (10.8)	0.58 (0.37-0.92)	0.02
Dominant model			0.75 (0.56-0.99)	0.05
Recessive model			0.66 (0.44-1.01)	0.05
Lymph node metastasis	Yes	No		
AA	109 (37.7)	186 (31.8)	1.00 (Ref)	
AG	150 (51.9)	312 (53.3)	1.22 (0.89-1.66)	0.22
GG	30 (10.4)	87 (14.9)	0.57 (0.35-0.95)	0.02
Dominant model			1.30 (0.96-1.76)	0.09
Recessive model			1.56 (0.99-2.44)	0.05

CRC, colorectal cancer; OR, odds ratio; CI, confidence interval.
† adjusted by age and gender.
